# Functional Breads with Encapsulated Vitamin C and Fish Oil: Nutritional, Technological, and Sensory Attributes

**DOI:** 10.3390/antiox13111325

**Published:** 2024-10-30

**Authors:** Angelo Uriho, Kaiwen Chen, Fanlin Zhou, Lingling Ma, Cheng Chen, Shuning Zhang, Jacob Ojobi Omedi, Weining Huang, Ning Li, Li Liang

**Affiliations:** 1 State Key Laboratory of Food Science and Resources, Jiangnan University, Wuxi 214122, China; 7210102910@stu.jiangnan.edu.cn (A.U.);; 2 School of Food Science and Technology, Jiangnan University, Wuxi 214122, China; 3 Guangzhou Puratos Food Co., Ltd., Guangzhou 511400, China

**Keywords:** functional bread, ascorbic acid, fish oil, retention, quality, flavor

## Abstract

The fortification of bread is considered an effective approach for improving its nutritional properties. However, the incorporation of free bioactive components into bread formulations may affect the overall quality of breads in different ways, depending on the sensitivity of bioactive components to baking factors. In this study, the incorporation of encapsulated vitamin C (ascorbic acid and its salts) and fish oil in breads was investigated for their stability and effect on bread quality. The combination of fish oil emulsions increased the retention of encapsulated ascorbic acid, calcium ascorbate, or sodium ascorbate in breads compared to that of the free non-combined vitamin. At the same time, the combination of vitamin gels increased the retention of docosahexaenoic acid (DHA) and decreased the lipid oxidation in breads compared to the non-combined encapsulated forms. The highest retention values of ascorbic groups, eicosapentaenoic acid (EPA), and DHA were about 70%, 88%, and 95% in breads after baking, respectively. There was the negative correlation between the ABTS radical scavenging capacity and peroxide value in breads. The specific volume of breads was improved by vitamin gels but reduced by fish oil emulsions. Their combination resisted individual impact on the specific volume of breads. The breads with combined ascorbic acid gels and fish oil emulsions showed similar textural properties to the control one. The functional bread with calcium ascorbate gel and fish oil emulsion had the highest moisture content of 45.87%. The inclusion of vitamin gels plus fish oil emulsion decreased free water but increased the bound water. Combining ascorbic acid gels with fish oil emulsions effectively reduced and masked the fishy flavor. The integration of encapsulation techniques and multi-nutrient fortification is proposed as an effective way to enhance the nutritional value and quality of functional bread through synergistic effects.

## 1. Introduction

Bread has been a fundamental staple food in many cultures, serving as a primary source of sustenance for centuries. Although white bread is a basic food of everyday human nutrition, its consumption in high quantities is implicated in the development of non-communicable diseases, such as obesity, heart disease, and diabetes [1,2]. The fortification of bread is considered an effective approach for improving its nutritional properties with safe and nutritionally adequate micronutrient and macronutrient supplies [3]. Additionally, fortification may have a positive impact on the sensory attributes and technological properties of bread, making it a viable approach to enhance the nutritional profile and consumer acceptance of breads [4,5].

Eicosapentaenoic acid (EPA) and docosahexaenoic acid (DHA) are primarily found in fatty fish. They can help reduce the risk of heart disease [6] and improve age-related neurodegenerative diseases, vision problems, and growth disorders in children [7,8]. Enriching bread with fish oil is a way to improve its nutritional quality and increase the intake of EPA and DHA [9]. However, polyunsaturated fatty acids (PUFAs) are labile to oxidation. Canola oil contains a higher content of PUFAs, leading to a higher production rate of peroxides in multigrain bread in comparison to olive oil. The addition of ascorbic acid decreased the lipid oxidation of canola oil but increased that of olive oil in multigrain bread [10].

Ascorbic acid is a water-soluble vitamin C with antioxidant activity. Calcium ascorbate and sodium ascorbate are used as a neutral mineral ascorbate. The inclusion of vitamin C in bread can help increase the overall vitamin content of the diet and address potential deficiencies, particularly in populations where the consumption of fruits and vegetables (natural sources of vitamin C) may be limited [11,12]. The antioxidant property of vitamin C can help prevent the oxidation of food ingredients [13], maintain the quality and extend the shelf life of foods, and prevent the degradation of n-3 PUFAs and certain vitamins (e.g., vitamin A and folate) [14,15,16].

Environmental factors, including pH, temperature, oxygen, light, and moisture, have a significant impact on vitamin C and PUFAs [11,17]. The main current challenge in the development of functional bread fortified with ascorbic acid and PUFAs is to maintain their stability during breadmaking processes [11,18]. The fortification with PUFA-rich fish oil may produce unpleasant fishy off-flavors and peroxides due to the inherent characteristics of fish oil and the oxidation of PUFAs [18]. The high baking temperature may cause the degradation of ascorbic acid and PUFAs, followed by the formation of secondary volatile products [19,20,21]. Encapsulation technology can provide a physical barrier to environmental factors during baking [4,12,22,23,24]. Fat-coated ascorbic acid in pup-loaves of white pan breads, spray-dried ascorbic acid microparticles with gum Arabic in biscuits and with xyloglucan in fish burgers, and microencapsulated PUFAs powder in whole wheat bread have been investigated to improve their stability [4,12,22,23,24]. Whey proteins possess functional properties, such as emulsification and gelation, and their assemblies are the potential carriers for encapsulating, protecting, and delivering bioactive components [25,26].

The combination of different antioxidants may produce a positive or negative effect on their bioactivity and stability [16,27,28,29]. PUFAs and vitamin C showed a synergistically therapeutic effect against methotrexate-intoxicated mice [30]. There is currently no work on their combined effect on bread’s nutritional and physicochemical properties, although many works have emphasized the fortification of bread with either vitamin C or omega-3 PUFA-rich oils [12,31,32]. Evaluating the nutritional and technological impact of novel elements that emerge after breadmaking is crucial in the context of creating inventive wheat-based bread. In this regard, the present work aimed to investigate the combined effects of encapsulated vitamin C (ascorbic acid and its salts) and omega-3-rich sablefish oil on the bread’s quality and the stability of the bioactive ingredients.

## 2. Materials and Methods

### 2.1. Materials

Fish oil (Sablefish, 18% EPA, 12% DHA) was obtained from Shanghai HOPE Industry Co., Ltd. (Shanghai, China). l-Ascorbic acid (AA), calcium ascorbate (CaA), and sodium ascorbate (NaA) were provided by Luwei Pharmaceutical Group Co., Ltd. (Zibo, China). Whey protein isolate (WPI) was provided by Agropur (Saint-Hubert, QC, Canada). Other ingredients used for making bread, such as sodium salt, sugar, and wheat flour, were obtained from local supermarkets (Wuxi, China). Other materials of analytical grade were purchased from Sino-Pharm CNCM Ltd. (Shanghai, China).

### 2.2. Preparation of Fish Oil Emulsions and Vitamin C Gels

Oil-in-water emulsions were prepared according to a previous report [33] with a minor modification. Exactly 20 g of fish oil was added into 180 g of 2% (*w*/*w*) WPI aqueous solution under shearing at 16,000 rpm and then passed through an ATS high-pressure homogenizer (ATS Engineering Inc., Brampton, ON, Canada) at 10 °C and 200 bar for three times. After preparation, the emulsion was either utilized immediately or stored at 4 °C for subsequent application in bread fortification.

Vitamin gels were prepared according to our previous report. The ascorbic acid gel was prepared by adjusting the heat-denatured WPI solution pH to 4 using a freshly prepared l-AA solution (1.42 M, pH 1.4). For calcium ascorbate and sodium ascorbate gels in 10 mL of a heat-denatured WPI solution, 500 µL of l-ACa (586 mM, pH 6.5) and 1460 µL of l-ANa (1261 mM, pH 7.2) were added to obtain a final concentration of 30 mM and 185 mM, respectively. All samples were adequately mixed and then stored at 4 °C overnight for gelation and further use.

### 2.3. Bread-Making Procedure

The fortified breads with free or encapsulated vitamin C (ascorbic acid or calcium ascorbate or sodium ascorbate) and fish oil (FFO and EFO) were prepared according to the recipes presented in Table S1 using the straight dough method [34]. Five types of bread, including wheat bread (Control), free vitamin bread (FAA, FCaA, and FNaA), encapsulated vitamin breads (EAA, ECaA, and ENaA), free fish oil bread (FFO), encapsulated fish oil bread (EFO), combined free ascorbic acid and fish oil (FAA+FFO, FCaA+FFO, and FNaA+FFO), and combined vitamin gel and fish oil emulsion breads (EAA+EFO, ECaA+EFO, and ENaA+EFO) were prepared. Except for the vitamin C gel, all weighed ingredients were initially mixed in a spiral mixer (Sinmag Bakery Equipment, Wuxi Co., Ltd., Wuxi, China) at a slow speed for 3 min and a fast speed for 1 min; then, the vitamin C gel was added at a slow speed for 3 min and a fast speed for 2.4 min. After the dough rested for 5 min, 90 g of the dough pieces were rounded and rested for 5 min. Subsequently, the dough was molded in baking pans, and the proofing process was conducted at a temperature of 38 °C with a relative humidity of 85% for 90 min. The baking process was performed in a preheated oven (Sinmag Machinery Co., Wuxi, China) with the top heating set at 170 °C and the bottom heating set at 210 °C for 17 min. The breads were analyzed after cooling for 2 h.

### 2.4. Retention of Vitamins and Polyunsaturated Fatty Acids

Ascorbic acid and its salts were analyzed using the AOAC Official HPLC method 2012.21 [35] with some modifications. Briefly, 20 mL of 4.5% metaphosphoric acid was mixed with 5 g of samples and centrifuged at 10 °C and 6000 rpm. The supernatant was passed through a 0.22 µm filter and injected into the HPLC system (Waters, Milford, MA, USA) using a 2998 PDA detector at 245 nm and a T3 column (5 µm, 46 mm × 250 mm) at 35 °C. The mobile phase was composed of milli-Q water, methanol, and formic acid at a volume ratio of 970:30:3 with a flow rate of 1 mL/min.

Lipid extraction was conducted following a previous method [36]. After 2 g of lyophilized bread powder was vigorously mixed with 1 mL of ethanol and then 5 mL of hexane, the mixture was centrifuged at 4 °C and 5000× *g* for 5 min, and the supernatant was evaporated using nitrogen. The methylation of fatty acids was conducted using a previous method [37]. The fatty acid methyl esters were then quantified using a model GC-2010 PLUS (Shimadzu Co., Kyoto, Japan) with a DB-WAX column (30 m × 0.25 mm, 0.25 μm) and with a flame ionization detection at 250 °C for both the injector and detector. The temperature started at 50 °C and was raised to 200 °C at a rate of 25 °C/min and then to 230 °C at a rate of 3 °C/min and maintained at that level for 23 min. The flow rates were 10.0 mL/min for helium as a carrier gas and 30 mL/min for nitrogen, 400 mL/min for air, and 40 mL/min for hydrogen as make-up gases, and the shunt ratio was 10:1. The retention of bioactive components in bread was determined as follows:

$$Retention(\%)=\frac{\mathrm{Bioactive}\mathrm{component}\mathrm{in}\mathrm{bread}}{\mathrm{Bioactive}\mathrm{component}\mathrm{in}\mathrm{nonproofed}\mathrm{dough}}\times 100$$

### 2.5. Specific Volume

The specific volume of the loaves was determined using the rapeseed displacement method (AACC method 10-05.01) [38]. The specific volume was calculated as the ratio of the volume and the mass of the breads.

### 2.6. Textural Profile Analysis

The textural profile was measured using a Texture Pro CT V 1.4 Build 17 (Brookfield Engineering Laboratory, Middleboro, MA, USA) equipped with an aluminum cylindrical probe of 36 mm diameter, which is the standardized one developed by the American Association of Cereal Chemists (AACC) for the measurement of bread crumb and other bakery products [39]. A double compression test was performed using the texture profile analysis test mode. The test parameters were as follows: pre-test speed of 3 mm/s, test speed of 1 mm/s, post-test speed of 5 mm/s, and trigger force of 5 g. Two slices from the middle of each piece of bread were crushed to half of their original thickness [40].

### 2.7. Moisture Content

The moisture content was determined by the weight difference before and after drying the samples in a vacuum oven according to the AACC 44-15.02 method [41]. Briefly, 2 g of crumb was weighed and oven-dried at 120 °C for 3 h. The moisture content of the crumb was evaluated in fresh bread and after 1, 3, 5, and 7 d of storage at 25 °C.

### 2.8. Water Distribution and Mobility Quantified by Low-Field Nuclear Magnetic Resonance (LF-NMR)

Proton distributions of bread crumbs during storage were measured using a 22.6 MHz low-field pulsed NMI20 (Shanghai Niumag Corporation, China) LF-NMR analyzer [42]. Briefly, 1.5 g of bread crumbs were taken from the center of bread slices after 0 and 7 days, placed into a 25 mm NMR tube, and sealed with polytetrafluoroethylene sealing tape to prevent water loss. Then, the polytetrafluoroethylene sealing containing bread was placed within a specialized tube measuring 30 mm × 200 mm for LF-NMR testing. The primary parameters were configured as follows: TW (time waiting) = 2000 ms, TE (time echo) = 0.5 ms, NECH (number of echoes) = 14,000, and NS (number of scans) = 2.

### 2.9. Electronic Nose Analysis and Consumer Evaluation

#### 2.9.1. Electronic Nose Analysis

The Heracles II, a high-speed gas chromatography electronic nose (Alpha M.O.S., Toulouse, France), comprises an HS100 autosampler for sampling, 18 metal oxide sensors, and MXT-5 and MXT-1701 columns connected to two flame ionization detectors to obtain a comprehensive fingerprint and to analyze the volatile compounds. Briefly, 3 g of sample in a sample bottle was sealed and then placed in a headspace autosampler. A single sample injection was performed for simultaneous analysis using two DB-5 and DB-1701 columns with contrasting polarities. The temperature started at 50 °C and increased to 80 °C at a rate of 1 °C/s and then to 250 °C at a rate of 2 °C/s, where it was maintained for 60 s. The carrier gas was helium at a rate of 1 mL/min. The detector temperature was set at 260 °C. The principal component analysis (PCA) uses two principal components to respond to the original variable information.

#### 2.9.2. Sensory Evaluation of Bread

The sensory evaluation of breads was conducted with the 9-point structured hedonic scale. Eight semi-trained panelists, comprising four male and four female students aged between 25 and 35 years, participated in the hedonic test. The panelists were familiar with the different bread attributes and were instructed to assess the overall acceptability, color, taste, texture, and aroma using a hedonic scale ranging from 1 to 9:1: dislike extremely, 2: dislike very much, 3: dislike moderately, 4: dislike slightly, 5: neither like nor dislike, 6: like slightly, 7: like moderately, 8: like very much, and 9: like extremely [43]. Each bread sample was sliced, randomly coded and presented to the panelists a minimum of 2 h after baking.

### 2.10. Oxidative Stability and Antioxidant Activity

#### 2.10.1. Peroxide Value

The peroxide value (PV) of fish oil was measured using the AOCS Official Method cd8-53 [44]. Lipid was extracted by vigorously mixing 2 g of freeze-dried bread powder with 1 mL of ethanol and then 5 mL of hexane. After the mixture was centrifuged at 4 °C and 5000× *g* for 5 min, 5 mL of the supernatant was mixed with 30 mL of glacial acetic acid/chloroform (3:2, *v*/*v*), and a saturated potassium iodide solution put in a 250 mL Erlenmeyer flask. Subsequently, the mixed solution was gently agitated for 1 min, followed by the addition of distilled water and saturated starch solution. The sodium thiosulfate solution was used to titrate the substance until it reached the state of being colorless. The PV was calculated by the following equation:

Peroxide value (%) = (V × N × 1000)/W

where V is the volume of the sodium thiosulfate solution required to titrate the sample, N is the normality of sodium thiosulfate solution, and W is the weight of the bread sample.

#### 2.10.2. Antioxidant Activity

Briefly, an ABTS radical solution containing 7.4 mM ABTS radical and 2.6 mM K_2_S_2_O_8_ was prepared by mixing the two compounds in the dark for 12 h. The diluted ABTS radical solution was combined with either samples or buffer at a volume ratio of 7:1. The mixture was kept in the dark for 6 min. The absorbance was measured at 729 nm using a UV-1800 UV–Vis spectrophotometer (Shimadzu Co., Tokyo, Japan). The radical scavenging activity was calculated as follows [45]:

$$\mathrm{Scavenging}\mathrm{capacity}(\%)=\frac{Ac-As}{Ac}\times 100$$

where Ac and As represent the absorbance of the radical plus buffer and the radical plus samples, respectively.

### 2.11. Statistical Analysis

All analyses were run in triplicate unless stated otherwise. The data were analyzed using a one-way analysis of variance (ANOVA) to compare the groups. Multiple comparisons were conducted to identify significant differences (*p* < 0.05) using the GraphPad Prism program 9.5 (GraphPad Software, Inc., San Diego, CA, USA). Origin 95E was used to analyze the data obtained from the fast gas chromatography electronic nose. A principal component analysis [46] was performed using the OmicStudio tools at https://www.omicstudio.cn/tool (accessed on 16 July 2024).

## 3. Results and Discussion

### 3.1. Retention of Ascorbic Acid, EPA, and DHA in Bread

**Figure 1:**
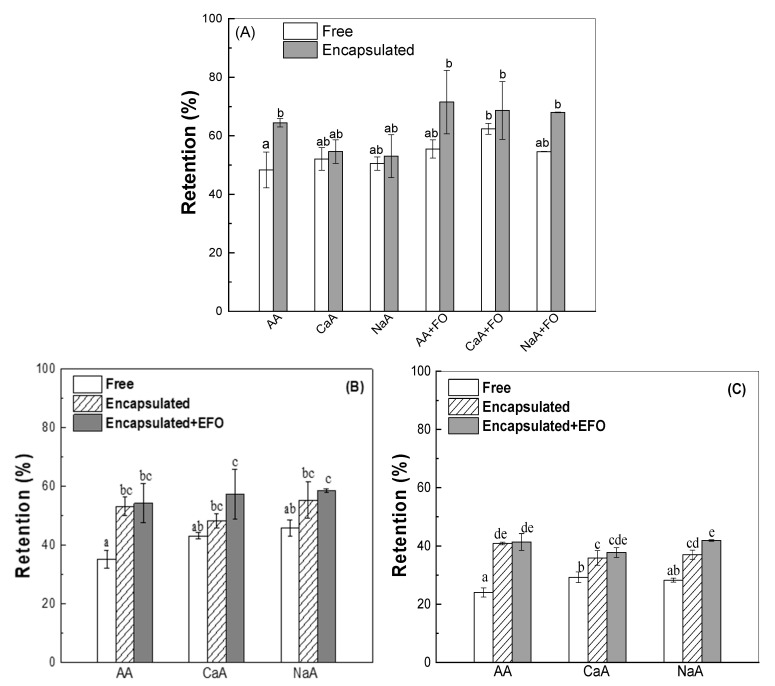
Retention of vitamin C in the bread for 2 h after baking (**A**), 1-day (**B**), and 3-day (**C**) storage at room temperature. AA: ascorbic acid; CaA: calcium ascorbate; NaA: sodium ascorbate. Different letters represent significant differences among the samples (*p* < 0.05).

Vitamin C is labile to degradation during bread baking [47]. The retention of ascorbic acid, calcium ascorbate, and sodium ascorbate was 50% in the breads after baking (Figure 1A). Being a cellular solid, the gluten network in the dough and bread provided a physical barrier to partially protect free vitamins against degradation (Figure 1). The encapsulation in the WPI cold-set gel (Figure S1) increased the retention of ascorbic acid to 64% in bread (Figure 1A). Similar results were obtained for the encapsulation with xyloglucan microparticles, which reduced the loss of ascorbic acid by 17% in tilapia fish burgers during baking [24]. However, the encapsulation in WPI gels did not affect the retention of calcium ascorbate and sodium ascorbate in bread (Figure 1A). The co-inclusion of fish oil did not affect the stability of free ascorbic acid and sodium ascorbate but improved that of free calcium ascorbate. It is noted that the addition of encapsulated fish oil (Figure S1) increased the retention of encapsulated ascorbic acid, calcium ascorbate, and sodium ascorbate to 69% in breads (Figure 1A). Therefore, the combination of encapsulation in WPI gels and the addition of encapsulated fish oil can improve the stability of ascorbic groups in breads during baking.

The retention of ascorbic groups was evaluated over a three-day storage duration. Figure 1B,C show that the retention of ascorbic acid and its salts decreased with the storage time. The loss of ascorbic acid is mostly due to oxidative processes [11,48,49]. During the three-day storage period, the retention of encapsulated vitamin C in breads was higher than that of free ascorbate groups. The vitamin retention was further improved by the presence of encapsulated fish oil. The retention of ascorbic acid and its salts was about 40% after storage for 3 d. Park et al. (2007) observed a lower degradation of ascorbic acid during storage in bread enriched with fat-coated ascorbic acid compared to the non-enriched control bread [48].

**Figure 2:**
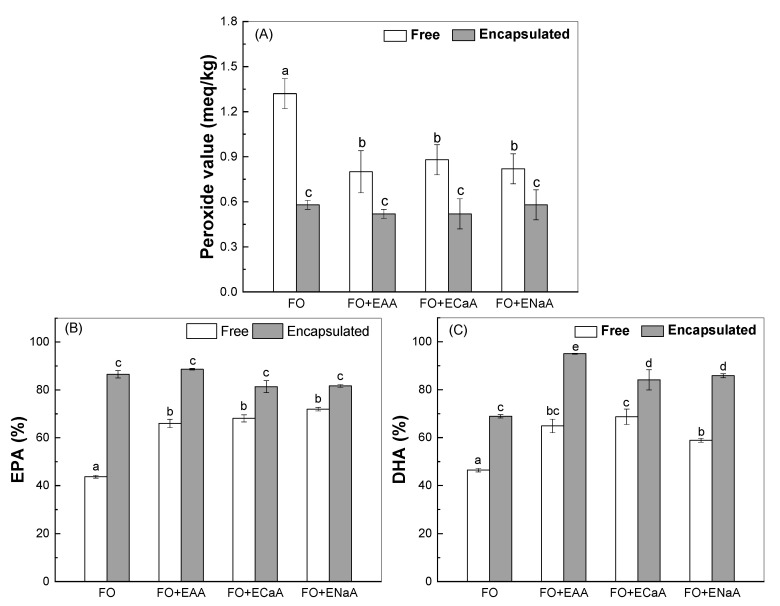
Peroxide value (**A**) and the retention of EPA (**B**) and DHA (**C**) in fish-oil-fortified breads without and with vitamin C. FO for fish oil, EAA for encapsulated ascorbic acid, ECaA for encapsulated calcium ascorbate, NaA for encapsulated sodium ascorbate. Different letters represent significant differences among the samples (*p* < 0.05).

The incorporation of fish oil into food products poses challenges due to its susceptibility to oxidation, leading to undesirable flavors and loss of nutritional value [50,51]. Figure 2A shows a peroxide value of 1.32 meq/kg in the bread with free fish oil. The retention of EPA and DHA was 45% when free fish oil was added to the bread (Figure 2B,C). The inclusion of encapsulated vitamin C decreased the peroxide value to around 0.82 meq/kg and increased the retention of DHA to 59–69% and the retention of EPA to 69% in the bread with free fish oil. The addition of ascorbic acid was utilized to prevent lipid oxidation in PUFA-fortified yogurt [52]. The encapsulation in WPI emulsions decreased the peroxide value to around 0.54 meq/kg, which was not affected by the addition of encapsulated vitamin C (Figure 2A). The encapsulation of fish oil in WPI emulsions increased the retention of EPA and DHA, respectively, to 86% and 69% in the bread without vitamin C (Figure 2B,C). The combination of encapsulated vitamin C did not affect the retention of EPA in the bread with encapsulated fish oil. However, the retention of DHA increased, respectively, to 95%, 84%, and 86% in the presence of encapsulated ascorbic acid, calcium ascorbate, and sodium ascorbate. Therefore, the combination of encapsulation in WPI emulsions and the addition of encapsulated vitamin C can improve the stability of fish oil in breads during baking.

### 3.2. Antioxidant Activity

In general, white bread has a low antioxidant activity, due to the decreased antioxidant content in white wheat flour caused by the refining process that eliminates the bran and germ of the wheat kernel, which are abundant in antioxidants [53]. ABTS radical scavenging activity of white breads was 29.17% (Table 1). The scavenging activity increased to 47.10% by the inclusion of free fish oil (Table 1), due to the presence of anti-oxidative PUFAs. The scavenging activities further increased to 61.79% by the inclusion of encapsulated fish oil (Table 1), attributed to the protection of WPI emulsion against lipid oxidation (Figure 2). The bread with both encapsulated ascorbic acid and encapsulated fish oil showed a higher radical scavenging capacity compared to non-combined and/or free forms (Table 1), due to the fact that the encapsulation increased the retention of ascorbic acid, EPA, and DHA during baking (Figure 1 and Figure 2). There was a negative correlation between the radical scavenging capacity (Table 1) and peroxide value (Figure 2A). Similar results were reported for whole-grain bread, since the accumulation of fatty acid peroxides may lead to a decrease in the antioxidative activity over time [54,55,56,57].

### 3.3. Quality Characteristics of Breads

#### 3.3.1. Specific Volume and Textural Properties

**Figure 3:**
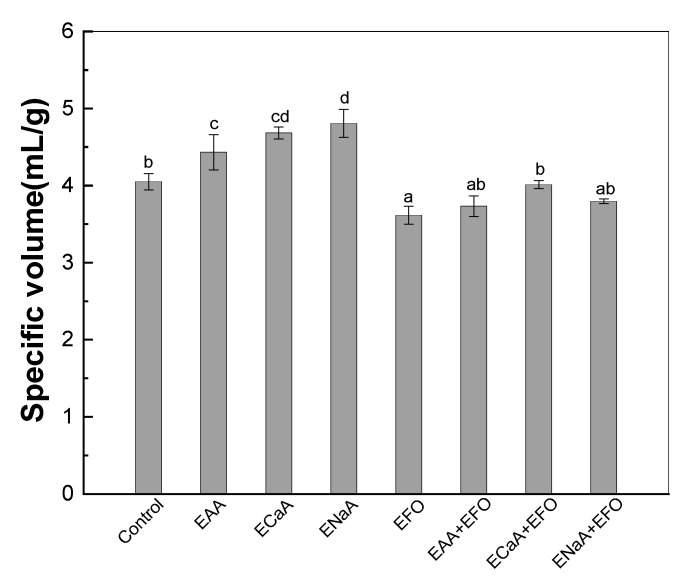
The specific volume of functional bread with encapsulated vitamin C and fish oil. EAA for encapsulated ascorbic acid, ECaA for encapsulated calcium ascorbate, ENaA for encapsulated sodium ascorbate, and EFO for encapsulated fish oil. Different letters represent significant differences among the samples (*p* < 0.05).

**Figure 4:**
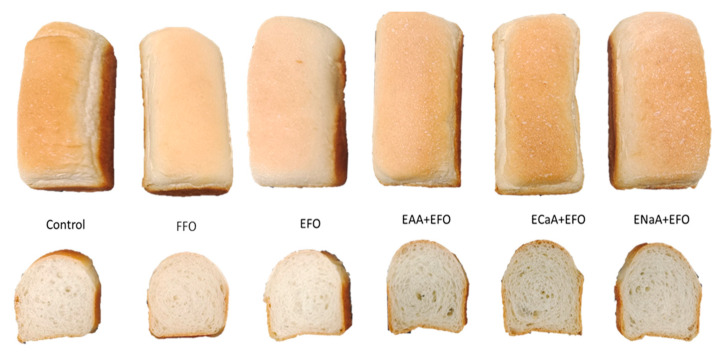
Bread samples. Control for white wheat bread; EFO for fish oil emulsion bread; EAA+EFO, ECaA+EFO, and ENaA+EFO for the breads fortified with combined fish oil and vitamin gels.

Figure 3 displays that the specific volume of the control breads was 4.05 mL/g. The specific volumes were higher in the presence of EAA, ECaA, and ENaA than in their absence (*p* < 0.05). A similar effect was previously reported, in that the addition of ascorbic acid increased the specific volume of bread to 20% [32,58]. Ascorbic acid and its derivatives are commonly used as baking improvers due to the fact that they increase dough strength and volume [11]. Ascorbic acid strengthens the dough protein network by creating disulfide bonds between peptides in the flour protein, leading to increased gas retention and bread volume [59,60]. Conversely, the addition of the fish oil emulsion decreased the loaf volume. Additionally, FFO and EFO bread crumbs were less porous compared to the control breads (Figure 4). This reduction effect may be attributed to the ability of DHA-rich fish oil to weaken the gluten structure, hence decreasing the specific volume of bread [31,61]. The breads with encapsulated fish oil and ascorbic acid showed a similar specific volume and porous structure to those of the control breads (Figure 3 and Figure 4). These results suggest that the combination of fish oil emulsion and ascorbic acid gel resists their respective impact on the specific volume of breads.

The textural characteristics of functional breads are presented in Table 2. The breads without and with encapsulated fish oil had similar textural characteristics. These results are inconsistent with a previous report that the bread hardness with nano-liposomal fish oil was lower than that of the control and free fish oil breads [9]. It has been reported that the addition of ascorbic acid reduced the hardness of both white wheat and whole wheat breads [58]. Similarly, the incorporation of AA gels significantly (*p* < 0.05) decreased the crumb hardness and chewiness with their values ranging, respectively, from 829 to 429 and from 51.30 to 31.20 (Table 2). The effect was also observed for ECaA and ENaA breads. The breads with a well-developed porous crumb structure may possess an enhanced gas retention capacity, leading to an increased specific volume and less crumb hardness [62]. A significant increase in cohesiveness was observed by the inclusion of encapsulated vitamin C. It was noted that the breads with combined ascorbic acid gels and fish oil emulsions showed similar textural properties to the control ones. Springiness was similar for all breads.

#### 3.3.2. Moisture Content and Water Distribution

**Figure 5:**
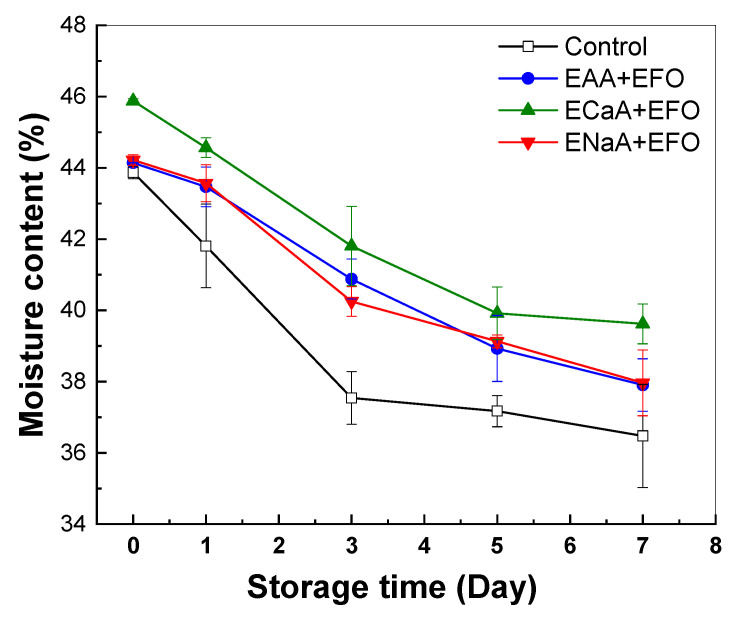
Moisture content in the crumb and crust of functional bread with combined ascorbic acid gels and fish oil emulsion during storage. EFO for encapsulated fish oil, EAA for encapsulated ascorbic acid, ECaA for encapsulated calcium ascorbate, and ENaA for encapsulated sodium ascorbate.

The moisture content of breads is a critical factor that directly influences its staling rate and shelf life [63,64]. It has been reported that the inclusion of PUFA-rich chia oil reduced water retention in bread after 14 days, which effect was inhibited by the encapsulation of the oil using a soy protein emulsion [65]. As shown in Figure 5, the moisture content of the control bread decreased rapidly during the first 3 days and remained 37% thereafter. The loss of moisture content was about 17% for the control bread. However, the loss of moisture content of functional breads was gradual and less than that of the control bread. The functional bread with calcium ascorbate gel and fish oil emulsion had a higher moisture content than the control bread and the bread with acid or sodium ascorbate plus fish oil emulsion. It has been reported that too much water loss during baking might result in a dry crust and early staling [66]. The decreased moisture loss during storage plays a crucial role in preserving crumb softness in breads, thus ensuring an anti-staling effect.

**Figure 6:**
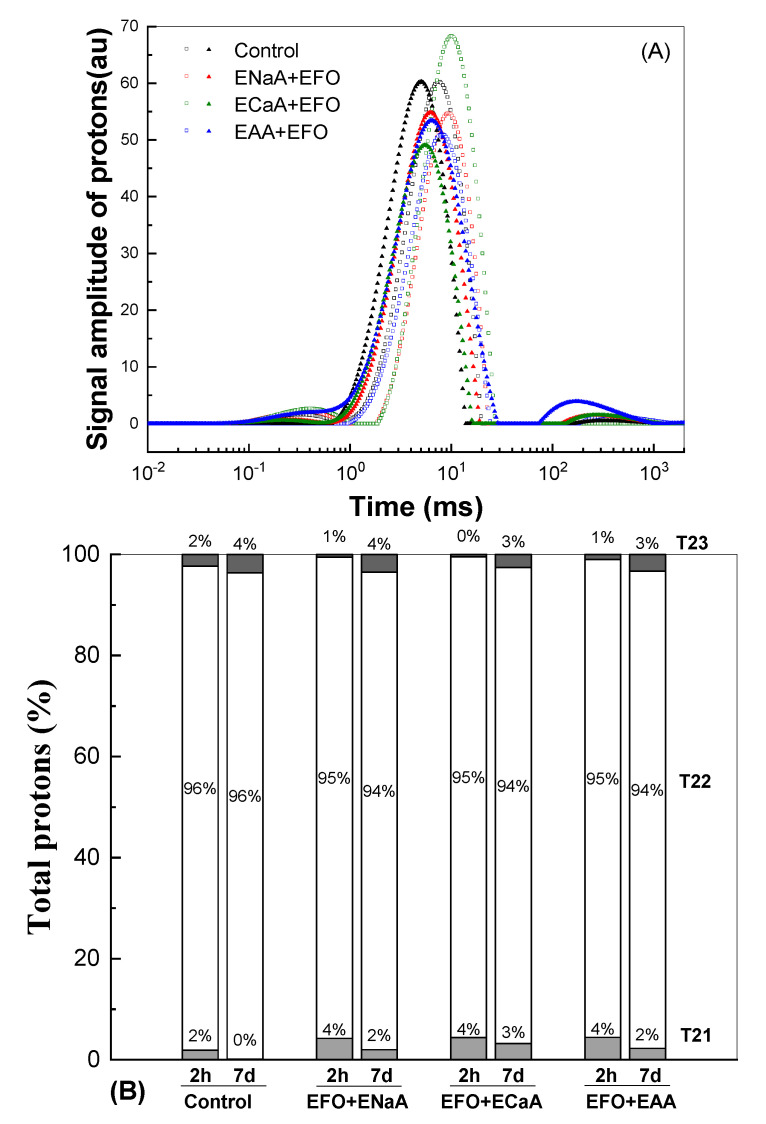
^1^HT2 distributions of the relaxation times (**A**) and relative abundance (**B**) in each proton population of the control and fortified breads after baking for 2 h (empty symbols) and 7 days (filled symbols).

LF-NMR was conducted to further confirm the water migration and distribution by the measurement of the relaxation time (T2). ^1^H T2 distributions are shown in Figure 6. Three ^1^H T2 populations were observed and named, from the shorter to the longer relaxation time, T21 (0–1 ms), T22 (1–25 ms), and T23 (70–1500 ms), respectively. The X-axis represents the water activity, and a shorter relaxation time is indicative of a lower degree of water freedom. The Y-axis represents the relative proportion of protons, and the peak area linearly represents each population’s proportional water content [67,68]. For the control bread, the T22 accounted for approximately 96% of all protons (Figure 6), making it the most abundant ^1^H population. The T21 and T23 populations represented approximately 2% of the total protons. In three different proton T2 populations, T21 accounted for bound water, which was trapped in the starch granules and gluten network; T22 accounted for immobilized water linked to retrograded starch, and T23 accounted for free water, with no interactions with other molecules [42,69]. The inclusion of vitamin C gels plus fish oil emulsion decreased the free water but increased the bound water (Figure 6). After 7 days of storage, a decrease in T21 but an increase in T23 were observed. It is suggested that bread samples with higher T22 and T23 proton populations exhibit a poor water-holding capacity [68,69]. Compared to the fortified breads (EAA+EFO, ECaA+EFO, and ENaA+EFO), the control bread showed a higher T23 proton population and a lower T21 proton population at day 7 (Figure 6). This was due to the incorporation of fish oil emulsion and ascorbic acid gels developed using whey protein isolate as the wall material, which increased the chemical nature of the proteins that hold water and prevented moisture loss [67,70].

#### 3.3.3. Aroma Evaluation of Breads Based on E-Nose and PCA Analysis

**Figure 7:**
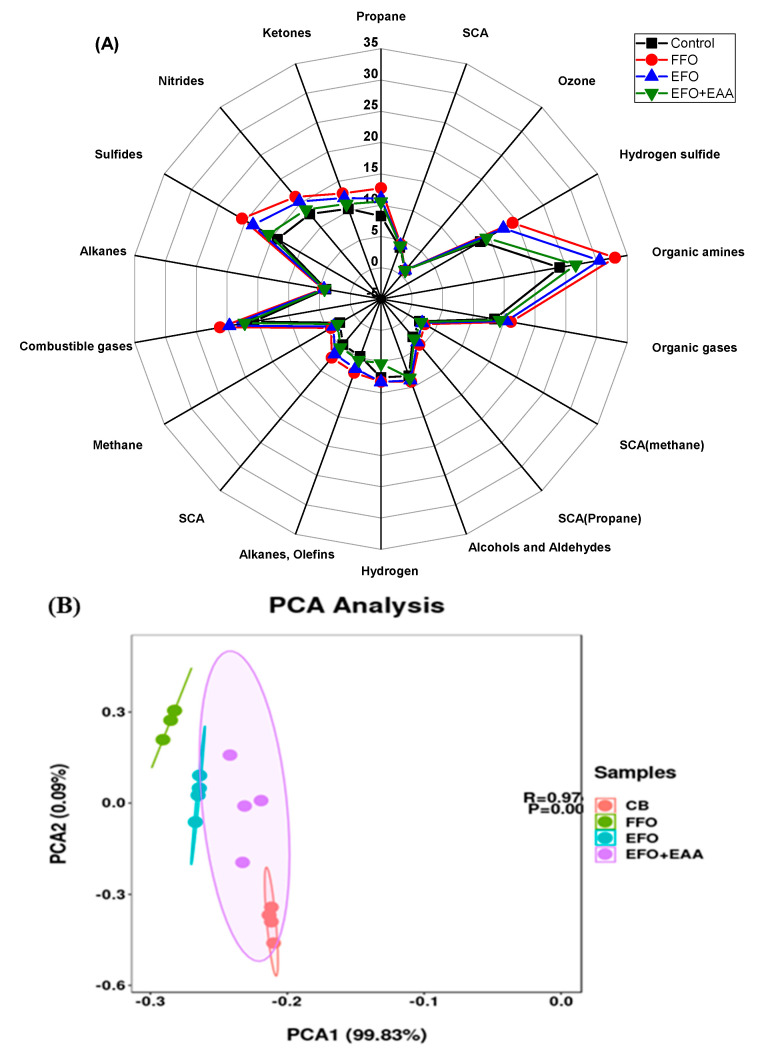

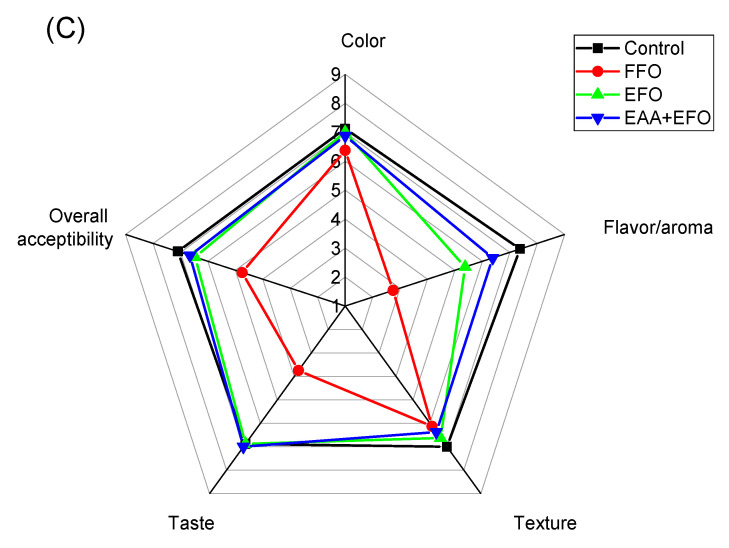
Electronic nose radar map (**A**) and principal component analysis (PCA, **B**) of flavor compounds in breads and consumer sensory evaluation of bread (**C**). CB: Control/wheat bread; FFO: free fish oil-fortified bread; EFO: encapsulated fish oil-fortified bread; EAA+EFO: the bread fortified with combined fish oil and ascorbic acid gel.

The flavor profile of breads can be analyzed using electronic nose (e-nose) technology for sensory evaluation [71]. The radar fingerprint chart of the volatile compounds obtained during the HERACLES electronic nose analysis of the functional bread is shown in Figure 7A. Each of the 18 sensors in the electronic nose exhibited a distinct level of sensitivity toward a certain type of odor or volatile compound [72]. The fishy odor (e.g., organic amine, ketones, and sulfides, Figure 7A) attributes were rated as highly intense in the free fish oil [73]. The encapsulation of fish oil in whey protein emulsions decreased the fishy odor of the functional breads (Figure 7A and Table S2), due to the masking effect against release and protective effect against oxidation (Figure 2A). Similarly, in the human sensory evaluation, the bread with the free fish oil was rated the lowest in aroma, taste, and texture, while the inclusion of the fish oil emulsion and/or fish oil emulsion mixed with ascorbic acid gels improved the taste, aroma, texture, and overall acceptability (Figure 7C and Table S3). Rathod and Kairam found that the double encapsulation of fish oil using soya lecithin and whey protein–sodium alginate beads helped mask its distinct flavor [74]. The combination of encapsulated vitamin gels further decreased the fish odor of functional breads. Jacobsen et al. reported that a large part of the earthy–musty off-odor compounds of silver carp slices were removed by marinating them in ascorbic acid under microwave drying [75].

A principal component analysis (PCA) was also used to evaluate the ability of the sensor array to distinguish between the different groups of target gases [76]. The first two principal components, PCA1 and PCA2, were utilized to indicate the individual sensor’s contribution to solving the categorization problem. Figure 7B represents 99.92% of the total variance, with PCA 1 and PCA 2 contributing 99.83 and 0.09% of the variance, respectively. The score plot exhibits distinct clusters and differentiation between the free fish oil bread and other breads, indicating significant differences in flavor attributes (*p* < 0.001). The odor profile distribution of the FFO bread and other breads exhibited a long distance (Figure 7A), and the fitting range was not crossed (Figure 7B), indicating that their aromas were different. However, the proximity between the control and EFO+EAA breads was closer than that between the control and FFO/EFO breads, suggesting that their overall flavor was also relatively similar in comparison with the FFO and EFO breads. The e-nose radar map (Figure 7A), PCA analysis (Figure 7B), and sensory evaluation (Figure 7C) of the flavor profile characteristics of breads were closely consistent. These findings suggest that combining ascorbic acid with fish oil can be effective in reducing or masking the fishy flavor.

## 4. Conclusions

The encapsulation of vitamin C (ascorbic acid and its salts) in WPI gel and omega-3-rich fish oil in WPI emulsions effectively protected them from degradation under baking conditions. The bread co-fortified with vitamin gels and fish oil emulsions showed the highest retention of ascorbic groups and ω-3 polyunsaturated fatty acids during baking, as well as a significant increase in antioxidant activity. On the other hand, the association between encapsulated fish oil and vitamin C improved the physicochemical properties and quality of bread, particularly in terms of specific volume, texture, shelf life, and sensory acceptance of the functional breads by masking the fishy flavor. These findings provide valuable insights into the synergistic improvements in bioactive ingredients in the production of baked products with functional quality.

## Figures and Tables

**Table 1:** Antioxidant activity of functional bread fortified with free and encapsulated fish oil without and with encapsulated vitamin C.

Bread Fortification	ABTS Radical Scavenging Activity (%)
Control	29.17 ± 1.49 ^a^
FFO	41.70 ± 3.39 ^b^
EFO	61.79 ± 0.61 ^cd^
EAA	59.14 ± 0.82 ^cd^
ECaA	55.65 ± 0.67 ^c^
ENaA	55.23 ± 1.12 ^c^
EFO+EAA	67.24 ± 0.92 ^d^
EFO+ECaA	64.93 ± 3.85 ^d^
EFO+ENaA	62.88 ± 0.61 ^d^
FFO+EAA	61.17 ± 0.49 ^cd^
FFO+ECaA	56.76 ± 0.31 ^cd^
FFO+ENaA	59.38 ± 1.54 ^cd^

Note: FFO, fish oil; EFO, encapsulated fish oil; EAA, ECaA, and ENaA, respectively, for encapsulated ascorbic acid, calcium ascorbate, and sodium ascorbate. Different letters represent significant differences among the samples (*p* < 0.05).

**Table 2:** Textural properties of functional breads fortified with encapsulated ascorbic acid and fish oil.

Sample	Hardness (g)	Chewiness	Cohesiveness	Springiness
Control	829 ± 69 ^b^	51.30 ± 12.30 ^b^	0.65 ± 0.01 ^ab^	9.19 ± 0.05 ^a^
EAA	429 ± 2 ^a^	31.20 ± 4.22 ^ab^	0.80 ± 0.02 ^b^	9.21 ± 0.15 ^a^
ECaA	297 ± 6 ^a^	20.40 ± 0.10 ^a^	0.80 ± 0.10 ^b^	12.23 ± 5.10 ^a^
ENaA	342 ± 10 ^a^	25.60 ± 0.30 ^a^	0.79 ± 0.03 ^b^	11.03 ± 2.95 ^a^
EFO	742 ± 84 ^b^	50.46 ± 9.06 ^b^	0.76 ± 0.07 ^b^	9.10 ± 0.27 ^a^
EAA+EFO	728 ± 98^b^	33.10 ± 11.46 ^ab^	0.72 ± 0.01 ^b^	9.45 ± 0.38 ^a^
ECaA+EFO	657 ± 31 ^b^	31.95 ± 7.45 ^ab^	0.76 ± 0.00 ^b^	10.02 ± 0.38 ^a^
ENaA+EFO	681 ± 37 ^b^	47.36 ± 3.42 ^b^	0.62 ± 0.02 ^a^	10.03 ± 2.26 ^a^

Control for white wheat bread; EAA for encapsulated ascorbic acid; ECaA for encapsulated calcium ascorbate; ENaA for encapsulated sodium ascorbate; EFO for fish oil emulsion. Different letters represent significant differences among the samples (*p* < 0.05), (Tukey HSD^a^).

## Data Availability

Data is contained within the article or Supplementary Materials.

## Supplementary Materials

The following supporting information can be downloaded at: https://www.mdpi.com/article/10.3390/antiox13111325/s1. Figure S1: Visual observation of cold-set gels of WPI with ascorbic acid, calcium ascorbate, and sodium ascorbate (left) and of WPI-stabilized fish oil emulsion (right); Table S1: The recipes used to prepare the breads with free and combined encapsulated fish oil and vitamins; Table S2: The mean sensor responses of the volatile compounds of breads; Table S3: Consumer sensory evaluation of the functional breads.
