# Supplementary material for: Functional Breads with Encapsulated Vitamin C and Fish Oil: Nutritional, Technological, and Sensory Attributes
Source: Antioxidants (Basel). 2024 Oct 30;13(11):1325. doi: 10.3390/antiox13111325 (PMC11590905; doi:10.3390/antiox13111325)
Supplement: Supplementary file 1 [file antioxidants-13-01325-s001.zip › antioxidants-3238832-supplementary.pdf]

Supplementary materials

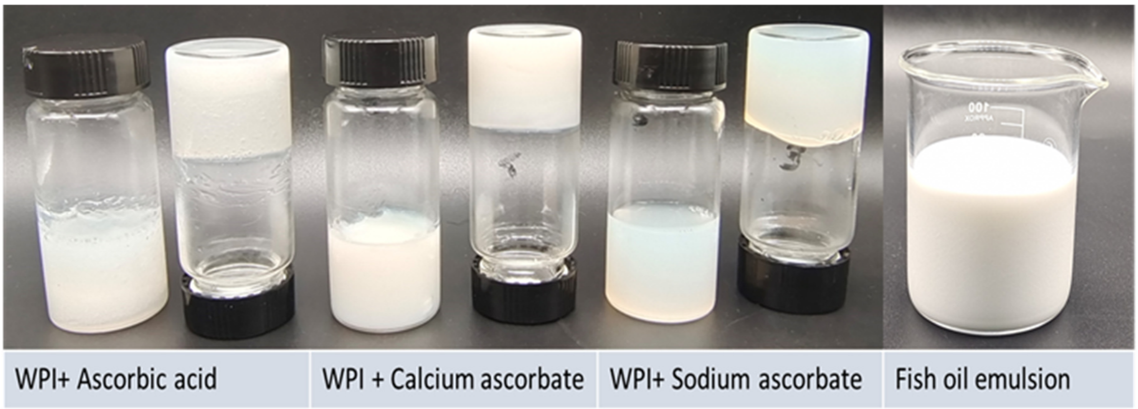

Figure S1 Visual observation of cold-set gels of WPI with ascorbic acid, calcium ascorbate and sodium ascorbate (left) and of WPI-stabilized fish oil emulsion (right).

Table S1 The recipes used to prepare bread with free and combined encapsulated fish oil and vitamin C

**Ingredients**	**CB**	**FAA, CaA, FNaA**	**FFO**	**EAA, ECaA, ENaA**	**EFO**	**FAA+FFO, FCaA+FFO, FNaA+FFO**	**EAA+EFO, ECaA+EFO, ENaA+EFO**
Wheat four (g)	300	300	300	300	300	300	300
Water (g)	180	180	180	180	70	180	70
Sugar (g)	24	24	24	24	24	24	24
Yeast (g)	4.5	4.5	4.5	4.5	4.5	4.5	4.5
Salt (g)	3	3	3	3	3	3	3
Fish oil (g)	-	-	12	-	-	12	-
Vitamin C (g)	-	0.25	-	-	-	0.25	-
Fish oil emulsion (g)	-	-	-	-	120	-	120
Vitamin C gel (g)	-	-	-	10.07		-	10.07

CB for control bread, FAA for free ascorbic acid, FCaA for free calcium ascorbate, FNaA for free sodium ascorbate, EFO for encapsulated fish oil, EAA, ECaA and ENaA respectively for encapsulated ascorbic acid, calcium ascorbate and sodium ascorbate.

Table S2 The mean sensor responses of volatile compounds of breads

Volatile compound	Control	FFO	EFO	EFO+EAA
Propane	8.26 ± 0.16[a]	12.71 ±0.08[d]	11.05 ± 0.17[c]	10.56 ±0.36[b]
Alcohols, aldehydes and SCA	3.80 ± 0.01[a]	4.29 ± 0.07[c]	4.14 ± 0.01[b]	4.06 ± 0.02[b]
Ozone	1.00 ± 0.00[a]	1.00 ± 0.00[a]	1.00 ± 0.00[a]	1.00 ± 0.00[a]
Hydrogen sulfide	13.33 ± 0.11[a]	19.18 ± 0.37[d]	17.59 ± 0.14[c]	14.49 ± 0.79[b]
Organic amines	23.99 ± 0.24[a]	32.98 ± 0.64[d]	30.49 ± 0.19[c]	26.61 ±0.96[b]
Organic gases	13.43 ± 0.06[a]	16.00 ± 0.20[d]	15.36 ± 0.05[c]	14.29 ± 0.33[b]
SCA (methane)	2.10 ± 0.01[a]	3.00 ± 0.13[c]	2.61 ± 0.05[d]	2.40 ± 0.06[b]
SCA(Propane)	2.93 ± 0.02[a]	4.52 ± 0.11[d]	4.03 ±0.07[c]	3.27 ± 0.19[b]
Alcohols, Aldehydes	8.06 ± 0.01[a]	9.02 ± 0.07[d]	8.81 ± 0.01[c]	8.42 ± 0.11[b]
Hydrogen	7.55 ± 0.44[b]	8.21 ± 0.69[b]	8.27 ± 0.82[b]	5.32 ± 1.44[a]
Alkanes and Olefins	4.74 ± 0.04[a]	7.52 ± 0.23[d]	6.82 ± 0.09[c]	5.37 ± 0.39[b]
SCA (methane, liquefied gas)	4.51 ± 0.03[a]	7.18 ± 0.30[d]	6.53 ± 0.10[c]	5.12 ± 0.39[b]
Methane	2.60 ± 0.02[a]	4.21 ± 0.20[d]	3.75 ± 0.02[c]	3.00 ± 0.18[b]
Combustible gases	15.66 ± 0.09[a]	20.96 ± 0.43[d]	19.61 ± 0.13[c]	17.13 ± 0.68[b]
Alkanes	3.94 ± 0.01[a]	4.40 ± 0.038[d]	4.27 ± 0.00[c]	4.18 ± 0.03[b]
Sulfides	14.12 ± 0.11[a]	20.63 ± 0.70[d]	18.65 ± 0.12[c]	15.73 ± 1.00[b]
Nitrides	12.74 ± 0.07[a]	16.27 ± 0.38[d]	15.33 ± 0.02[c]	13.68 ± 0.54[b]
Ketones	10.34 ± 0.05[a]	12.91 ± 0.27[d]	12.19 ± 0.00[c]	11.17 ± 0.39[b]

The unit of volatile compounds is ppmv. Control for white wheat bread; FFO for fish oil bread; EFO for fish oil emulsion bread; EAA+EFO, bread fortified with combined fish oil and ascorbic acid gel; SCA, short chain alkanes. Different letters represent significant differences among the samples ($p < 0.05$).

Table S3 Consumer sensory evaluation of the functional breads

Sense	Control	FFO	EFO	EAA+EFO
Color	7.12 ± 1.45^{a}	6.38 ± 1.84^{a}	7.00 ± 1.92^{a}	6.88 ± 1.72^{a}
Flavor/aroma	7.38 ± 0.91^{b}	2.75 ± 2.25^{a}	5.38 ± 2.19^{b}	6.38 ± 1.30^{b}
Texture	7.00 ± 1.85^{a}	6.12 ± 1.88^{a}	6.62 ± 1.06^{a}	6.38 ± 1.50^{a}
Taste	6.88 ± 1.64^{b}	3.75 ± 2.49^{a}	6.88 ± 1.72^{b}	7.00 ± 1.41^{b}
Overall acceptance	7.09 ± 0.89^{b}	4.75 ± 1.46^{a}	6.47 ± 1.53^{ab}	6.66 ± 1.34^{b}

Control for white wheat bread; FFO for fish oil bread; EFO for fish oil emulsion bread; EAA+EFO, bread fortified with combined fish oil and ascorbic acid gel; Different letters represent significant differences among the samples ($p < 0.05$).
